# Assessing the Feasibility of a Text-Based Conversational Agent for Asthma Support: Protocol for a Mixed Methods Observational Study

**DOI:** 10.2196/42965

**Published:** 2023-02-02

**Authors:** Rafael A Calvo, Dorian Peters, Laura Moradbakhti, Darren Cook, Georgios Rizos, Bjoern Schuller, Constantinos Kallis, Ernie Wong, Jennifer Quint

**Affiliations:** 1 Dyson School of Design Engineering Imperial College London London United Kingdom; 2 Department of Computing Imperial College London London United Kingdom; 3 Faculty of Medicine National Heart & Lung Institute Imperial College London London United Kingdom

**Keywords:** conversational agent, chatbot, health, well-being, artificial intelligence, health education, behavior change, asthma

## Abstract

**Background:**

Despite efforts, the UK death rate from asthma is the highest in Europe, and 65% of people with asthma in the United Kingdom do not receive the professional care they are entitled to. Experts have recommended the use of digital innovations to help address the issues of poor outcomes and lack of care access. An automated SMS text messaging–based conversational agent (ie, chatbot) created to provide access to asthma support in a familiar format via a mobile phone has the potential to help people with asthma across demographics and at scale. Such a chatbot could help improve the accuracy of self-assessed risk, improve asthma self-management, increase access to professional care, and ultimately reduce asthma attacks and emergencies.

**Objective:**

The aims of this study are to determine the feasibility and usability of a text-based conversational agent that processes a patient’s text responses and short sample voice recordings to calculate an estimate of their risk for an asthma exacerbation and then offers follow-up information for lowering risk and improving asthma control; assess the levels of engagement for different groups of users, particularly those who do not access professional services and those with poor asthma control; and assess the extent to which users of the chatbot perceive it as helpful for improving their understanding and self-management of their condition.

**Methods:**

We will recruit 300 adults through four channels for broad reach: Facebook, YouGov, Asthma + Lung UK social media, and the website Healthily (a health self-management app). Participants will be screened, and those who meet inclusion criteria (adults diagnosed with asthma and who use WhatsApp) will be provided with a link to access the conversational agent through WhatsApp on their mobile phones. Participants will be sent scheduled and randomly timed messages to invite them to engage in dialogue about their asthma risk during the period of study. After a data collection period (28 days), participants will respond to questionnaire items related to the quality of the interaction. A pre- and postquestionnaire will measure asthma control before and after the intervention.

**Results:**

This study was funded in March 2021 and started in January 2022. We developed a prototype conversational agent, which was iteratively improved with feedback from people with asthma, asthma nurses, and specialist doctors. Fortnightly reviews of iterations by the clinical team began in September 2022 and are ongoing. This feasibility study will start recruitment in January 2023. The anticipated completion of the study is July 2023. A future randomized controlled trial will depend on the outcomes of this study and funding.

**Conclusions:**

This feasibility study will inform a follow-up pilot and larger randomized controlled trial to assess the impact of a conversational agent on asthma outcomes, self-management, behavior change, and access to care.

**International Registered Report Identifier (IRRID):**

PRR1-10.2196/42965

## Introduction

### Asthma and Digital Health Interventions

It is estimated that 334 million people worldwide have asthma, which, in some countries, is 15%-20% of the population [1]. Despite effective treatments and extensive efforts, the UK death rate from asthma is the highest in Europe. Moreover, 65% of people with asthma in the United Kingdom do not receive the professional care they are entitled to (including a yearly review, feedback on how to use their inhaler correctly, or an asthma action plan). One of the reasons for this is believed to be poor self-assessment of risk by those with asthma. According to the charity Asthma + Lung UK, the illness is often not taken seriously enough. For example, 1 in 6 people in the United Kingdom do not know or are unsure if the condition can be fatal [2].

Mobile health interventions can help address these gaps in knowledge, self-assessment, and basic care. Mobile technologies can provide just-in-time, anywhere approaches to self-care or mixed mode care. They have been shown to be cost-effective in a number of health domains, and according to a recent systematic review, 87% improved adherence and 53% demosnstrated improved health outcomes [3]. Most often they are used to deliver clinical information, but efficacy has also been shown for providing elements of basic care and health risk feedback as well as supporting behavior change through personalized dialogue with conversational agents (CAs; as reviewed in [4-6]). For example, in the asthma domain specifically, Rhee et al [7] described an automated SMS text messaging system able to interpret conversational English SMS text messages, particularly around asthma symptoms. The system was tested by 15 adolescent-parent dyads, the results of which indicated that the messaging system was well received, easy to use, and convenient. More recently, Kadariya et al [8] tested kBot, a chatbot supporting pediatric asthmatic patients, integrated as part of an Android app. kBot was tested by clinicians and researchers (but not patients). Another study [9] described MAX, a CA focused on helping children learn about asthma and better use of their inhalers. MAX interacted with patients through SMS text messages and did not require a dedicated app. This single-arm feasibility study recruited patients’ family members in primary and secondary care (again patients were not included) and showed that using a CA can lead to improved cognitive and behavioral skills. These studies show the promise of this new digital health care approach and ways that it can complement primary and secondary care. What they do not contribute is a better understanding of what drives users to engage with such tools.

### The Collaborative Design Process

The larger project surrounding the study described in this protocol aims to develop a text-based automated CA to provide asthma risk assessment and follow-up support for improving asthma self-management. The design process entails development of the conversational scripts (conversational design) for the chatbot and technical development of the CA. The design process has involved patients, asthma nurses, and doctors (including 2 respiratory specialists) from the earliest stages. Stakeholder input was gathered in a variety of ways, including a stakeholder design workshop at the launch of the project, sample dialogue sessions with patients and doctors, a large patient survey (n=1257), and regular reviews of the chatbot and its conversational texts by 3 asthma nurses and 3 doctors.

Regular stakeholder involvement has shaped the content, format, and conversational style of the chatbot. A first critical design decision arising from stakeholder consultations was the choice to provide the chatbot through the popular WhatsApp platform rather than through a new dedicated app. The advisory team based the decision on WhatsApp’s lower barrier of entry for users. For example, because WhatsApp is used by a majority of people with asthma (85% in our survey) and does not require computer expertise or high-end hardware, it is more easily accessible for users than downloading and using a new app. Easier access is especially important for helping harder-to-reach populations.

Design and development of the chatbot was informed by a user research survey (N=1257) with adults with asthma. The survey explored prospective user preferences with respect to an asthma chatbot and perceptions around chatbots and technologies for health more broadly. Survey results indicated that 53% of respondents were very or somewhat interested in an asthma chatbot, yet only 7% had used an asthma app. The survey complemented previous surveys commissioned by Asthma + Lung UK, which also provide evidence for user interest in digital technologies for self-care [10].

Design of the conversational dialogues for the chatbot were also informed by qualitative research activities including analysis of a series of asthma conversations held between patients and clinicians. The complete results of the survey and qualitative studies have not yet been published. The conversational design was also informed by existing literature, input from our steering committee, and survey results.

Privacy is of utmost importance in health conversations. While a chatbot using WhatsApp may not be Health Insurance Portability and Accountability Act compliant, conversations are encrypted “end-to-end,” meaning that the messages are not readable by WhatsApp. Asthma + Lung UK and many other organizations already provide support, for example, by asthma nurses communicating through this channel.

### Exacerbation Risk Models

Machine learning experts and epidemiologists within the project have so far developed two risk models. One is based on the Clinical Practice Research Datalink (CPRD) data sets, while the other implements a machine learning model trained on voice recordings of individuals with or without a self-reported asthma diagnosis.

For the first model, we used the unique medical records of 1,277,745 patients and logistic regression. These were divided into three parts to implement temporal validation: 948,934 (74.3%) in the training sample; 244,588 (19.1%) in the testing sample; and 84,223 (6.6%) in the validation sample.

The voice-based risk model is intended to detect the level of asthma control and is based on the sound of their voice. It is based on a similar approach used successfully for COVID-19 diagnosis. The asthma risk model has been initially trained with a database available to researchers upon request and after signing a use agreement [11]. The database contains multilingual voice recordings and other metadata, including age, smoking status, and health information such as COVID-19 or other pulmonary diseases. Voice recording data submitted by participants will help to improve the voice risk model.

**Figure 1:**
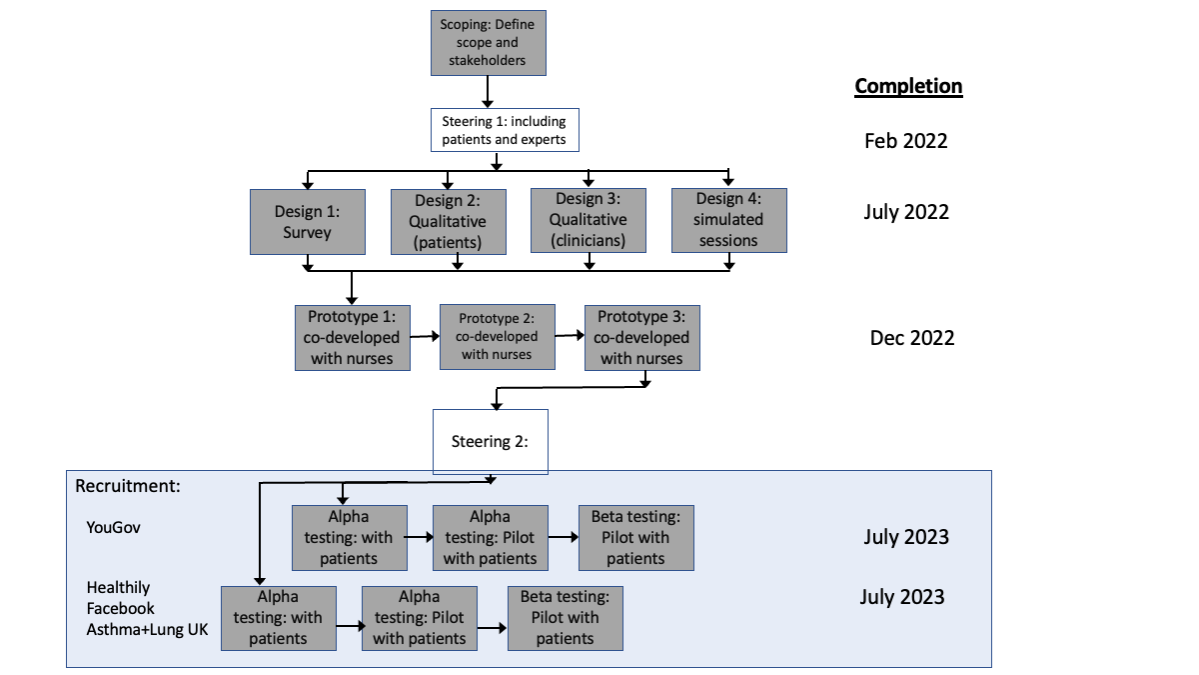
Design engineering process. The highlighted area describes the scope of this feasibility study. The rest shows the iterative and multistakeholder design process so far.

The findings from developing these models will be reported when completed and are out of scope for this feasibility protocol. Overall, the design engineering process, described in Figure 1 is similar to the systematic development process for patient decision aids described by Coulter et al [12].

### Engagement With Health Care and Technology

Self-determination theory (SDT) has been used as a theoretical framework for assessing efficacy of both health care environments and technologies. For example, it has been shown that a health care environment can improve health outcomes by supporting the basic psychological needs of patients [13]. Likewise, SDT can be used to predict the conditions for motivation to engage with a technology [14] as well as user satisfaction with that technology [14] (D Peters and R Calvo, unpublished data, 2023). As such, the conditions for need-supportive design of technology and health care environments from the SDT literature [15,16] will be incorporated into the design of the chatbot. Accordingly, we will include a set of SDT measures [14] in the postquestionnaire.

Of the different categories of clinical communication identified by Street Jr and Epstein [17], *information exchange* is the most readily used with CAs. In other words, CAs are typically used for collecting data from users by asking questions (eg, gathering a clinical history) and for disseminating information to them (eg, general information about an illness or how it can be managed). Other functions described in the Street Jr and Epstein [17] model, such as helping clients with managing uncertainty or decision-making, are less common because they rely more on open-ended exchanges. As such, exploring the feasibility of supporting such decision-making, including through a mix of closed- and open-ended dialogues, is among the aims of our project.

Person-centered care focuses on “understanding the patient as a unique human being” [18]. As such, our CA will focus on customizing conversation as much as possible, for example, by asking questions to understand the user’s unique asthma triggers, lifestyle factors, and risk of exacerbation, and then tailoring advice accordingly. Critically, this information gathering and sharing will be delivered as part of a natural dialogue that can adapt to user responses and convey information in gradual conversational chunks, where the user can control the pacing and focus. These features differentiate it from a more traditional questionnaire and results approach.

For example, the risk assessment form on the Asthma + Lung UK website presents the user with a series of form questions and then presents a collection of information based on their results. The form is easy to fill out and the results are personalized and carefully presented to make the information as useful as possible. However, the result is still similar to an information sheet. This relies on the user reading all the information and being able to absorb a large amount of mixed information at once. In contrast, we anticipate that the added adaptive agility, familiarity, and progressive disclosure of information that occurs as part of a conversation may provide benefits to understanding, retention, and behavior change, at least for some groups. In this initial feasibility study, we will include a number of self-report measures with respect to understanding and behavior change to begin to test this possibility. However, to compare the two approaches rigorously in a future study, we hope to arrange a controlled experiment comparing the chatbot with an online questionnaire to determine the advantages of each.

### Chatbot Content and Features

**Figure 2:**
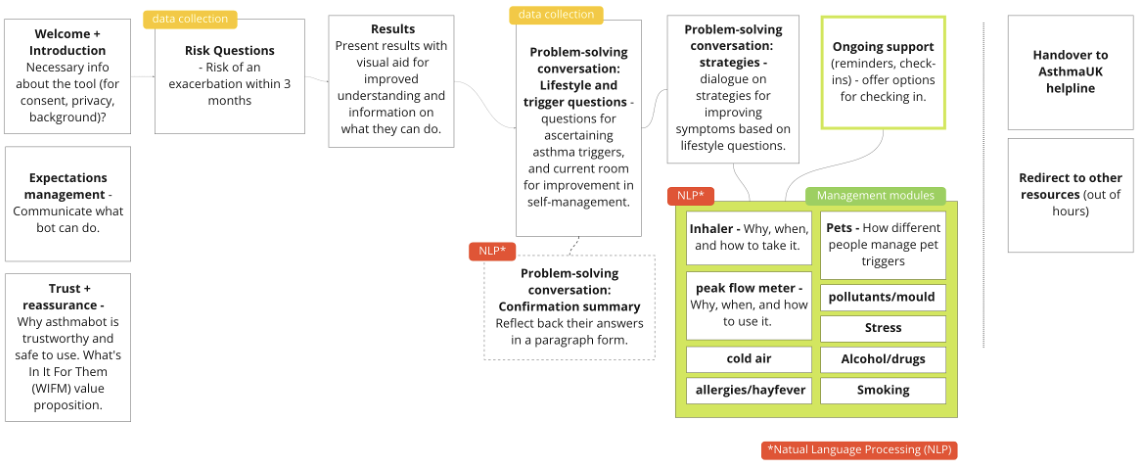
Diagram of conversational components. Boxes marked “NLP” include dialogues that may be generated based on a synthesis of user text input or that may be triggered by open-text input.

**Figure 3:**
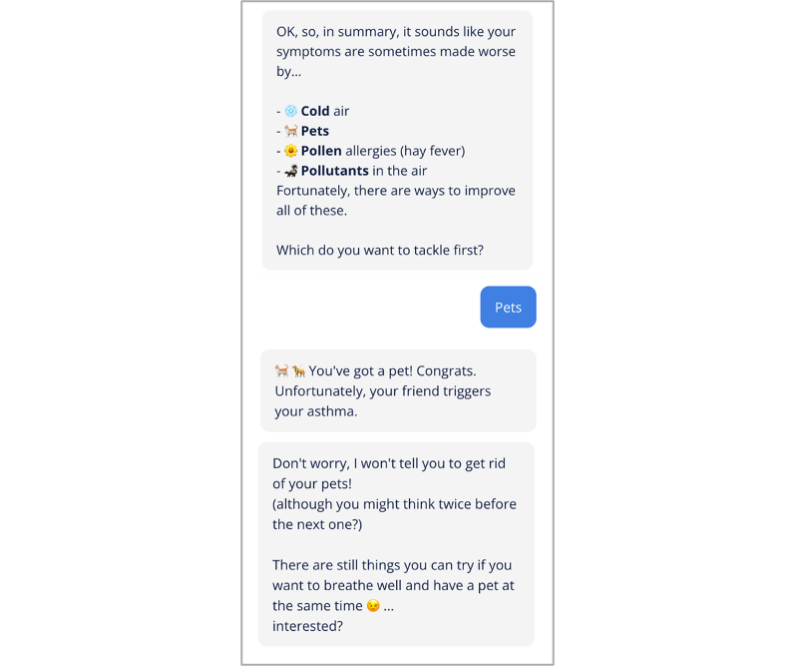
Example dialogue excerpt from the problem-solving component.

Following best practices for person-centered care technologies [19], the chatbot system described herein has been designed collaboratively with asthma patients, asthma nurses, and specialist doctors. Based on this approach, the chatbot was designed with a number of dialogue components each representing a topic about which the chatbot can engage in a conversation with the user (Figure 2). In short, the bot assesses a user’s risk of having an asthma exacerbation and then provides guidance on how to reduce this risk. The risk reduction guidance process proceeds by first supporting the user to identify their triggers and presenting strategies for managing their specific triggers based on their specific situation (see the example dialogue excerpt in Figure 3). The strategy dialogues will include referrals to Asthma + Lung UK or National Health Service content (eg, inhaler technique videos, smoking cessation clinic sites) with direct links to these resources. We will track click-throughs on these links as an indicator of engagement and impact.

The various dialogues can be triggered either by arriving at them naturally at the end of another dialogue (eg, the risk assessment component leads users to the problem-solving conversation component), selecting them from a list of options (like a menu, users can choose to skip the risk assessment and jump to a conversation about their asthma triggers), or through open-text input (a user can type “what about my dog?” and the system will respond with the dialogue on strategies for managing pet triggers). Free text is processed by a dedicated natural language processing server. For example, the chatbot asks for a description of the perceived triggers and maps it to specific categories. If none are automatically identified, a multiple-choice menu is offered.

## Methods

### Recruitment

Participants for the alpha and beta phases will be patients with an asthma diagnosis, as described above. Participants should be 18 years or older, able to communicate fluently in English, and willing to use WhatsApp on their mobile phones.

Participants will be compensated for their time (which involves trying the chatbot at least once and filling out a pre- and postquestionnaire on day 1 and 28, respectively) with a £10 (approximately US $12) voucher. The research study questionnaires will be provided in Qualtrics, separate from the chatbot so as not to interfere with the experience of the chatbot itself.

A total of 300 individuals will be recruited in three waves. Only approved phone numbers (of people who have consented to the study) will be able to interact with the chatbot.

Recruitment will be done over four channels:

1. YouGov: 120 adults with diagnosed asthma will be recruited through YouGov. We expect roughly 10% of the cohort who participated in the survey or 20% of those who expressed interest in the chatbot.
2. Facebook: 60 participants with self-reported asthma will be recruited through Facebook’s advertising engine.
3. Healthily: 60 participants with self-reported asthma will be recruited through Healthily (a medically approved self-care platform). Healthily will advertise the study in the “Asthma Hub” accessed by people with, or interested in, asthma. The timeline will be as the one above. People using Healthily are interested in self-care and pay £29.99 (approximately US $36.70) per year to access it.
4. Asthma + Lung UK social media: 60 participants with self-reported asthma will be recruited through social media channels managed by our partner organization.

The social media channels will direct people to a web page that will screen for inclusion criteria. Those who meet the criteria will be invited to participate, asked for informed consent, and directed to the prequestionnaire and then to the chatbot in WhatsApp on their mobile phones.

Recruitment and chatbot use will be phased to allow for iterative improvements. We will start recruitment through YouGov. The first batch of 20 individuals will use it for 1 week, and their feedback and engagement measures will be used to inform a new iteration of improvements. The same will be done with a second batch of 20 people. We refer to these groups as alpha testing in Figure 1. The third and final batch of 260 people will be drawn from the remaining recruitment channels and will use the system for 28 days. We refer to this group as beta testing in Figure 1.

### Procedure

We estimate that the average conversation will take less than 15 minutes. Participants will be sent randomly timed messages once a week to invite them to track their asthma risk during the period of the study. After the data collection period (28 days), participants will respond to survey items related to the quality of the interaction. At day 28, they will be sent a final reminder inviting them to complete the exit questionnaire with a link to a Qualtrics form. A follow-up WhatsApp message sent 3 months later will ask users to report if they had an asthma exacerbation within the last 3 months (since they used the bot), and they will have one additional month (which means 4 months from using the bot) to provide this information on exacerbations.

For clarity, if the project were to start on January 1, the first group would finish on January 7. Feedback would take a week to address, and the second group would start on January 14, finishing on January 21. The third batch would start on February 1 and finish on March 1; a follow-up message asking about any asthma exacerbations would be sent on June 1 (allowing them to respond until July 1).

The outcome variables and associated measures are presented in Table 1.

**Table 1:** Outcome variables and associated measures.

Variable	Measure
Acceptability, usability, and satisfaction of the chatbot, including the risk assessment dialogue and follow-up dialogues on asthma triggers and management strategies for improvement of asthma control	We will combine the following standard technology satisfaction measures: Task completion (% complete risk assessment process vs drop-out rate) Net Promoter Score question: “how likely are you to recommend the chatbot to someone else with asthma on a scale from 0 to 10” User satisfaction questions for each dialogue topic (eg, “Did you measure your risk using the chatbot? How useful did you find this feature?”) Open-ended questions on what worked well and what did not, and how the chatbot could be improved
Motivational quality, engagement, and user experience in relation to basic psychological needs	Motivation, Engagement, and Thriving in User Experience questionnaire [14]
Alignment between expected and calculated risk, and whether the calculated result changes risk self-assessment	Self-report questions on risk score expectation vs result and trust in result
Changes to asthma understanding, attitudes, and behaviors following use of the app	Self-report questions on changes to understanding, attitudes, and behaviors related with asthma self-management
Self-reported level of asthma control and symptoms, including changes over time	The Asthma Control Questionnaire [20] will be asked at the beginning and end of the study
Patterns of engagement and satisfaction for different types of users, particularly those who do not access professional services and those with poor asthma control	Analysis of pre- and postquestionnaires and additional self-report questions on use of professional services (eg, “when was the last time you spoke to a GP about asthma”)

### Ethics Approval

The study protocol will be submitted to the Imperial College London research ethics committee. The user research survey informing the chatbot development was approved by the Imperial College London Ethics committee 21IC7403.

### Data Management and Plan

Transcripts (and data extracted from them) will be stored in a secure database. Responses to the pre- and postquestionnaires will be stored on Qualtrics’ servers.

Each user’s WhatsApp number will be recorded by the system to ensure that the user has provided consent to take part in the research study, track engagement by the same participant over time as well as link engagement patterns to questionnaire data, and improve the user experience (eg, the bot can then store the answers users gave in a previous session and does not start from scratch each time the conversation is returned to). Emails will be stored to send the £10 (approximately US $12) electronic voucher rewards.

Quantitative analysis of dialogue transcripts, use statistics, and questionnaire data will explore patterns and seek findings in relation to the variables and measures in Table 1.

### Analysis Plan

We seek to answer the following questions:

- Are there differences among the dialogue topics with regards to engagement? If so, which topic is most engaging? Do participants engage more, less, or equally with the artificial intelligence–enhanced motivational interviewing informed dialogues versus the health education branching dialogues? Does engagement with any topic correlate with better health or technology satisfaction outcomes?
- Although the goal of the study is not assessing the efficacy of the intervention, will the group’s asthma control improve at the end of the 4 weeks? A future randomized controlled trial will explore the efficacy with appropriate power and rigor, but this study might give initial indications of effect sizes.
- Did the iterative development approach improve outcomes? To test if the alpha and beta predict different effects, and assuming they have nonnormal distributions, we will use the nonparametric Kruskal-Wallis test.

## Results

This study was funded in March 2021 and started in January 2022. We plan to publish the results from the user research survey and qualitative studies in the near term and results from the feasibility study in the following term. We have already developed the CA and several risk assessment algorithms. The conversational design and algorithms will be improved and validated with the data collected during phase 1, started in September 2022.

Phase 2 of this feasibility evaluation will start recruitment in January 2023. The anticipated completion of the study is July 2023.

We hope the results will identify the extent to which the chatbot is a viable product that users find valuable, in particular for those who do not access professional services and whose asthma is poorly controlled. We expect that the level of engagement of all users will be positively related to how well the system supports their psychological needs.

## Discussion

People living with asthma need support to improve the accuracy of their risk self-assessment and asthma self-management, increase their access to professional care, and ultimately reduce asthma attacks and emergencies. Digital health technologies like apps offer new ways to address these but are often not used. In our survey, only 7% of people with asthma had used an asthma app. On the other hand, most use messaging platforms; in particular, 85% use WhatsApp. This has led us to develop a chatbot intervention to be delivered through WhatsApp that estimates the risk of an asthma exacerbation and provides recommendations on how to manage it better.

Our proposed study will evaluate the feasibility of this WhatsApp chatbot, with a particular focus on improving our understanding of the drivers and obstacles of engagement. While the chatbot has been designed and developed using the best practices available, such understanding will allow for better health chatbots in the future.

With respect to study limitations, since this is only a feasibility study, we are recruiting a relatively low number of participants (N=300). As such, we do not expect to find sex or age differences within the current sampling, and we do not expect to measure statistically significant improvements in asthma control over the 28 days. Furthermore, as we rely on self-report of diagnosed asthma (including having been prescribed a blue reliever inhaler that is only available through prescription), we could get some responses from someone who incorrectly answered the screening questionnaire.

In the future, findings from this study will be validated as part of a randomized controlled trial to determine whether the chatbot can measurably improve health outcomes. All these results will inform the development process of future chatbots in the health domain.

## Abbreviations

CA: conversational agent
CPRD: Clinical Practice Research Datalink
SDT: self-determination theory

## Data Availability

Risk models were created with Clinical Practice Research Datalink data and are available from the UK’s Medicines and Healthcare products Regulatory Agency, as described at [21]. Pending ethics approval and to support scientific transparency, dialogue data will be made available upon request to the corresponding author.
